# Fermentation Enhancement of Methanogenic Archaea Consortia from an Illinois Basin Coalbed via DOL Emulsion Nutrition

**DOI:** 10.1371/journal.pone.0124386

**Published:** 2015-04-17

**Authors:** Dong Xiao, Su-Ping Peng, En-Yuan Wang

**Affiliations:** 1 State Key Laboratory of Coal Resources and Safe Mining, China University of Mining and Technology, Xuzhou, Jiang Su province, China; 2 State Key Laboratory of Coal Resources and Safe Mining, China University of Mining and Technology, Beijing, China; 3 Faculty of Safety Engineering, China University of Mining and Technology, Xuzhou, Jiang Su province, China; University of Oklahoma, UNITED STATES

## Abstract

Microbially enhanced coalbed methane technology must be used to increase the methane content in mining and generate secondary biogenic gas. In this technology, the metabolic processes of methanogenic consortia are the basis for the production of biomethane from some of the organic compounds in coal. Thus, culture nutrition plays an important role in remediating the nutritional deficiency of a coal seam. To enhance the methane production rates for microorganism consortia, different types of nutrition solutions were examined in this study. Emulsion nutrition solutions containing a novel nutritional supplement, called dystrophy optional modification latex, increased the methane yield for methanogenic consortia. This new nutritional supplement can help methanogenic consortia form an enhanced anaerobic environment, optimize the microbial balance in the consortia, and improve the methane biosynthesis rate.

## Introduction

Methane is an important fuel for energy production worldwide, and due to growing markets, demand is increasing. Many methods have been used to extract methane from oil deposits and coal fields. If certain nutrients are injected into a coalbed, methanogenic consortia can metabolically convert some organic compounds into methane. Microbially enhanced coalbed methane (MECoM) technology is a procedure for producing methane and secondary biogenic gas to increase the methane content in mining.

Nearly all previous studies regarding coal biogasification have focused on using a microbial community to convert coal into methane, carbon dioxide, or other organic molecules in surface bioreactors [1–6]. Research on methane reservoirs has shown that microbial gas is typically located at shallow depths in thermally immature coals (at a vitrinite reflectance R_0_<0.6%), where formation waters exhibit relatively low salinity (<2 mol/L Cl^-^) and low ${SO}_{4}^{2-}$ concentrations (<10 mmol/L). The infiltration of dilute meteoric waters into these systems plays an important role in the transport of nutrients, the dilution of coalbed formation waters, and the stimulation of methongen growth.

**Fig 1:**
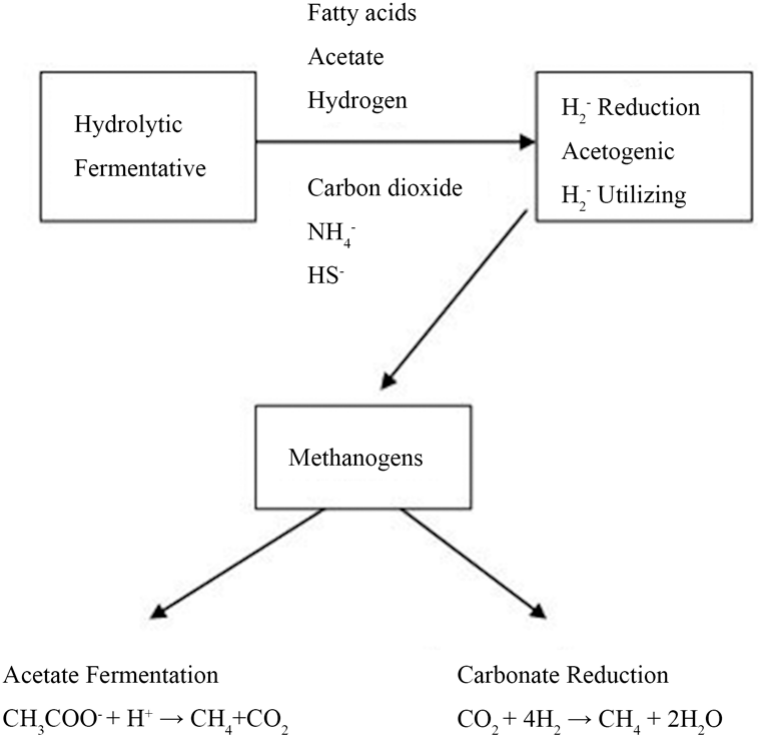
Generalized flow diagram for anaerobic decomposition of organic matter and generation of methane. Hydrolytic, hydrogen-reducing, acetogenic, and hydrogen-utilizing bacteria provide the organic compounds metabolized by methanogens.

Most organic compounds in coal cannot be directly used as nutrition for methanogens. Three functionally different trophic groups of bacteria (Fig 1) are required to convert organic material to methane [7–9]: (1) hydrolytic fermentative bacteria, (2) syntrophic acetogenic bacteria, and (3) methanogenic bacteria.

To achieve a greater yield of secondary biogenic gas in an Illinois basin coalbed, the nutritional components have been studied for the growth of, and stimulation of biomethane production in, methanogenic consortia [10,11]. The biomethane productivity of a methanogenic community can be improved if the culture conditions within the coal seam environments of the coalbed are improved. Dystrophy optional modification latex (DOL) contains sulfanilic acid, fatty acid oil, amide fat polymers, and other inorganic constituents. However, these emulsions are unstable. A DOL emulsion can improve the organic compound makeup of coal slurries and change the surface tension of the coal cleat system. The objective of this paper is to study microbial methane yields in the presence of DOL emulsions.

## Materials and Methods

### Source of coal and groundwater

The coal and groundwater samples used in this study were collected from two coalbeds in an Illinois basin. Coal samples were obtained, via drilling, from Gateway Mine, and formation water samples were collected from the Gateway gas well. Gateway Mine, in Randolph County near Coulterville, Illinois (GPS coordinates 38.181057,-89.656591), opened in March 2005 and started production in July of that year. The mine has 6 million tons of recoverable coal reserves. In 2013, the mine produced 2.8 million tons of steam coal [12]. The samples were collected by the Mining and Miner Engineering Center of Southern Illinois University Carbondale. The field studies did not involve endangered or protected species, and only small sample quantities were used.

**Fig 2:**
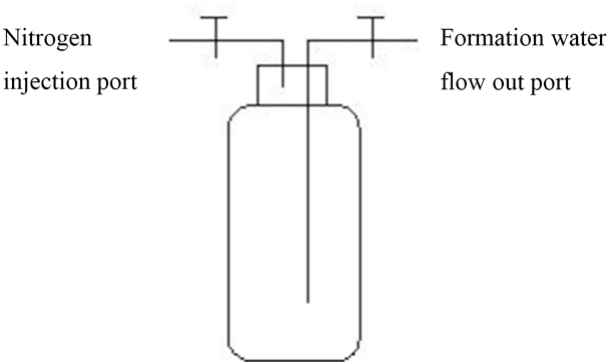
Barotropic anaerobic formation water storage jar.

The coal samples were sealed under nitrogen in sterile stainless steel jars (manufactured by Thermo Scientific Oxoid). The coal was safeguarded with nitrogen during sieve classification. A 2,000 g 10-to-15 mm-diameter fraction was collected and sealed in sterile wide-mouth polypropylene jars (manufactured by Thermo Scientific Oxoid) with nitrogen protection and stored at 4°C. Formation water samples of 5,000 mL were stored in sterile barotropic anaerobic jars (manufactured by Thermo Scientific Oxoid) (Fig 2) at 4°C. Nitrogen was used to increase the gas pressure at the top of the jar when extracting the formation water for sampling. In this manner, the groundwater was maintained under anaerobic and aseptic conditions.

### Culture

Two types of culture nutrition media based on the Tanner medium [13] for culturing strict anaerobes were used with modifications. The final concentrations of the compounds in the control culture nutrition medium (#1; g/L) were as follows: NaHCO_3_, 0.20; NH_4_Cl, 1.00; NaH_2_PO_4_, 1.30; KCl, 0.50; MgSO_4_•7H_2_O, 0.20; CaCl_2_•5H_2_O, 0.10; yeast extract, 0.50; resazurin, 0.10. The concentrations of DOL emulsion (g/L) were as follows: C_17_H_35_COONa, 46.50; R_1_SO_2_OR_2_, 28.50. The final concentrations of the compounds in the second nutrition medium (#2; g/L), which also contained the DOL emulsion, were as follows: NaHCO_3_, 0.20; NH_4_Cl, 1.00; NaH_2_PO_4_, 1.30; KCl, 0.50; MgSO_4_•7H_2_O, 0.20; CaCl_2_•5H_2_O, 0.10; C_17_H_35_COONa, 1.30; R_1_SO_2_OR_2_, 0.80; yeast extract, 0.50; resazurin, 0.10. Yeast extract was produced by Fisher BioReagents. C_17_H_35_COONa was produced by Alfa Aesar, and other chemicals were supplied by Acros Organics.

Distilled water was autoclaved at 121°C for 45 minutes to sterilize and remove any dissolved oxygen. The culture nutrition solutions were prepared in 500-mL flasks with distilled water and mixed by a magnetic mixer for 2 hours at 60°C. Then, the culture nutrition solutions were mixed at a 1:1 volume ratio with ground water and agitated by a magnetic mixer for another 2 hours at room temperature. Nitrogen was used to maintain the nutrition solutions under anaerobic conditions. The final pH of nutrition medium #1 was 6.40, and the final pH of nutrition medium #2 was 8.21.

Coal samples (200±1 g) and 700 mL of medium were used in the experiments. Anaerobic conditions were ensured in the flasks using the gas displacement method. The gas replacing process was monitored by a carbon dioxide sensor monitor in real time. Nitrogen was used to fill the air space above the flask at the beginning of the experiments. The flasks were placed in a water bath at 35°C and were cultured for 25 days. To examine the influence of the DOL emulsion on methanogenic consortia culture, medium #1 was used as the initial medium to culture the microbial community for the first 5 days. Then, medium #2 was used to replace medium #1. Nitrogen protection was used to ensure that no oxygen was introduced.

### Gas analysis

Real-time methane and carbon dioxide monitor systems were used in the experiments, including an IR15TT-R gas sensor and an IR-EK2 data receiver (produced by E2V company). The sensor uses two active gas channels for the simultaneous detection of methane and carbon dioxide. The measuring range for both methane and carbon dioxide was 0 to 100% by volume. The minimum resolution for methane was 0.05% within a measuring range from 0 to 5%, and 1% within a measuring range from 0 to 100%. For carbon dioxide, the minimum resolution was 0.5% within a measuring range from 0 to 100%. The sensor also monitored the temperature of the mixed gas. The IR-EK2 data receiver connected the IR15TT-R sensor to a computer. This component received, compiled, and transmitted real-time data from the IR15TT-R to the computer via a standard USB signal. The update rate and averaging time of these measurements were 180 s.

**Fig 3:**
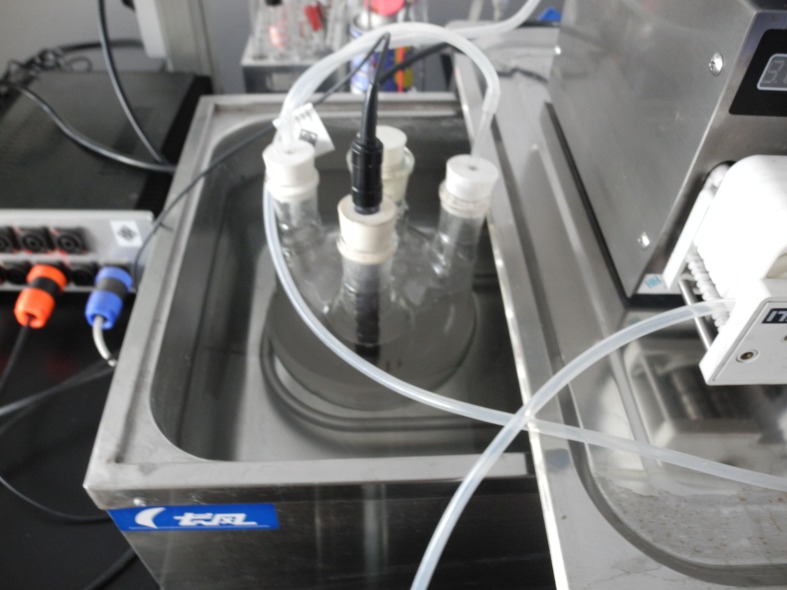
Three-neck flask. The left port was used for medium injection and sampling. The middle port functioned as the gas monitor port. The right port was used to monitor the pH.

Pure carbon dioxide, methane, and nitrogen were used to calibrate the sensor according to the manufacturer’s recommendations. Pure nitrogen was first used to initialize and create a baseline for both channels (methane and carbon dioxide). Pure methane was used to calibrate for 100% methane and 0% carbon dioxide. Pure carbon dioxide was used to calibrate for 100% carbon dioxide and 0% methane. Finally, pure nitrogen was used again to calibrate the zero level for both channels. Mixtures of 20% methane and 80% carbon dioxide, 50% methane and 50% carbon dioxide, and 80% methane and 20% carbon dioxide were used to test the sensor’s linear character. To obtain the best response time, the sensor was placed in the middle port of a three-neck flask (Fig 3).

### pH monitoring

The pH of their environment can influence the metabolism of methanogenic consortia. A lower pH can enhance the intrinsic activity of the microbes and the aqueous solubility of the coal [14,15]. At the same time, some microbes will produce hydrogen ions during the production of methane from organic matter. Thus, the microbial environmental conditions can be assessed via pH measurements.

A pH110 pH meter, which is produced by Oakton, was used in the experiments to monitor pH changes in the nutrition solutions. The pH110 meter can be used to monitor the real-time pH. The meter has a resolution and accuracy of ±0.01 pH units and an update frequency of 30 s.

### Microscopy

Fresh groundwater and fresh and replaced nutrition solutions were examined with an Olympus microscope (BX41) at 400× and 1000× magnification. The groundwater and coal samples were observed under non-sterile conditions. Microscopy was used to examine the nutrition solutions and to estimate the microbial consortium quantity under sterile conditions.

## Results

### Methane yield for a methanogenic consortium with nutrition solution #1

**Fig 4:**
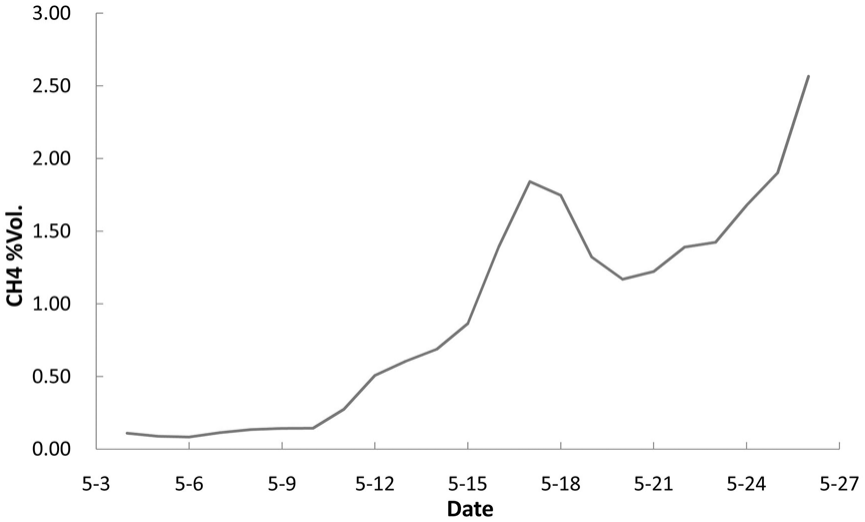
Methane volume concentration changes in a methanogenic consortium cultured with nutrition solution #1 at 39°C and pH 6.40. The contents of the solution were (in g/L) NaHCO_3_, 0.20; NH_4_Cl, 1.00; NaH_2_PO_4_, 1.30; KCl, 0.50; MgSO_4_•7H_2_O, 0.20; CaCl_2_•5H_2_O, 0.10; yeast extract, 0.50; resazurin, 0.10.

Nutrition solution #1 was a standard nutrition that used as a control culture medium which did not contain the DOL emulsion. The experimental results indicated that a minimum incubation time was required for the initial methanogenic consortium culture (Fig 4). As the culture developed, the methane concentration increased. However, the methane production rate of the methanogens was not ideal under these conditions because it was based on the hydrolytic and fermentative bacterial degradation of the organic matter in the coal [16–18].

**Fig 5:**
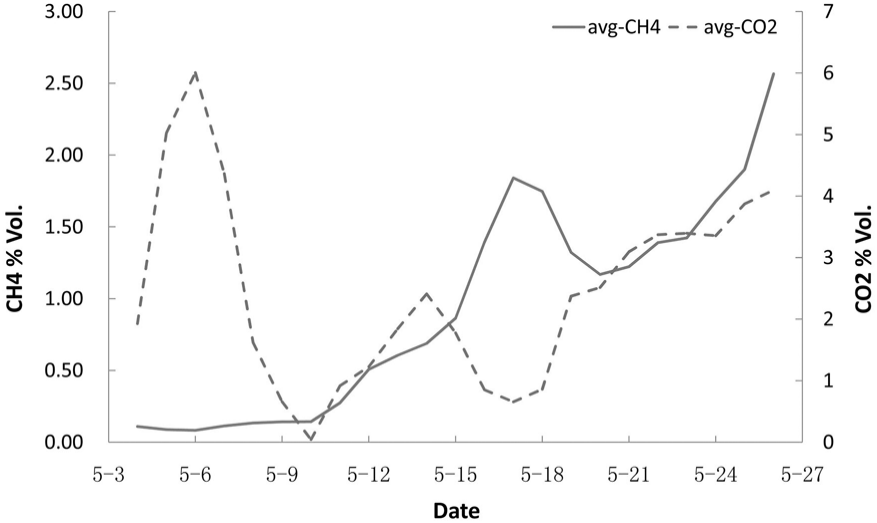
Methane and carbon dioxide concentration changes in a methanogenic consortia cultured with nutrition solution #1 at 39°C and pH 6.40.

Carbon dioxide is an important intermediate in the coal fermentation process and the main carbon source of methanogens. Thus, it is important to monitor the concentration of carbon dioxide in mixed gas as it affects the stability of methanogenic consortia. Initially, the carbon dioxide concentration increased more rapidly than the methane concentration under the conditions described above (Fig 5). This phenomenon indicates a higher reproductive rate for the hydrolytic bacteria compared to the methanogenic bacteria. The metabolic activity of the methanogens quickly consumed the available carbon dioxide. For this reason, the carbon dioxide production rate limited the growth and metabolism of the methanogenic bacteria and therefore limited the production of methane. Thus, the microbial balance between the methanogenic and hydrolytic bacteria consortia needs to be optimized to efficiently produce the desired methane. The different growth rates and balance between the two systems are thus important factors that require close monitoring.

### Methane yield for a methanogenic consortium with nutrition solution #2

Nutrition solution #1 has been widely used in anaerobic bacterial culture. Nutrition solution #2 contained 2.8% of the DOL emulsion by volume. The DOL emulsion is a mixed liquid that helps maintain equipment lubrication during mining. The effects of the DOL emulsion on the activity of the methanogenic consortia were examined in this experiment.

During the first six days, the methane yield remained at a low level. On the fifth culture day, nutrition medium #2 was used to replace nutrition medium #1. After this, the methane yield increased to a high level. The highest rate of methane production in this process was 40.32 mL/g·day. The high rate persisted for approximately 11 hours. This result indicates that nutrition solution #2 provided a better methane yield than nutrition solution #1 under the same environmental conditions.

**Fig 6:**
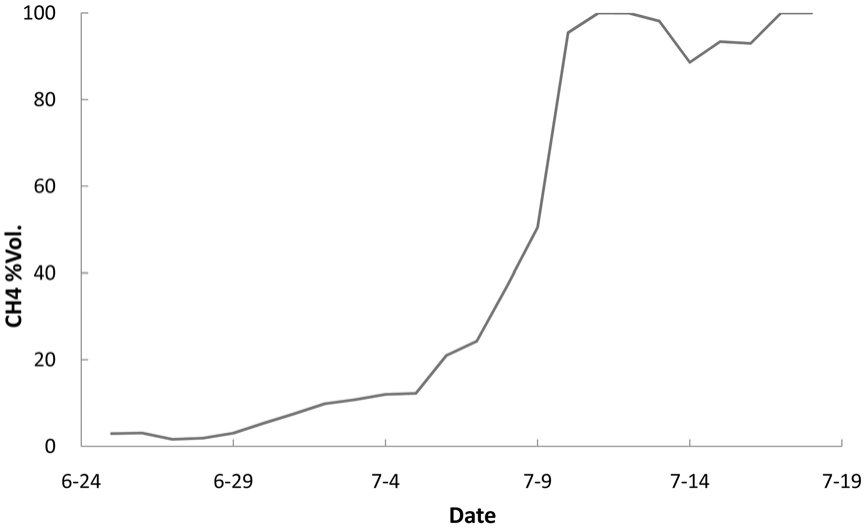
Methane concentration changes for a methanogenic consortia cultured with nutrition solution #2 at 39°C and pH 8.21. The contents of the solution were (in g/L) NaHCO_3_, 0.20; NH_4_Cl, 1.00; NaH_2_PO_4_, 1.30; KCl, 0.50; MgSO_4_•7H_2_O, 0.20; CaCl_2_•5H_2_O, 0.10; C_17_H_35_COONa, 1.30; R_1_SO_2_OR_2_, 0.80; yeast extract, 0.50; resazurin, 0.10.

In both experiments, an initial cultivation period was required before any significant methane production was observed. The first experiment required approximately 7 days, and the second experiment required approximately 6 days, i.e., methane production began 1 day earlier. For both experiments, the methane yield rates were not stable. A volatility factor was present during the methane production period. However, during the second experiment, no carbon dioxide was detected in the produced gas. This result indicates that the coalbed microbial community in solution #2 was more balanced than the community in solution #1 (Fig 6).

**Fig 7:**
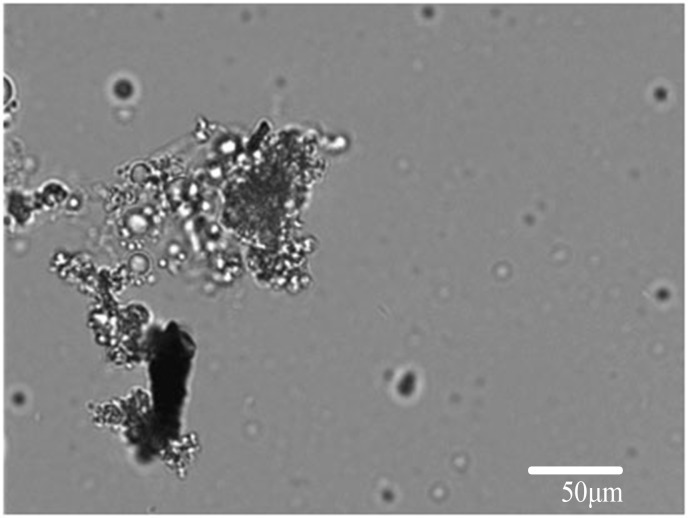
DOL emulsion oil adsorption on the coal surface under fluorescence microscopy at 400x magnification.

The DOL emulsion was unstable in the coal cleat system. A few compounds precipitated and were adsorbed at the coal surface (Fig 7). This precipitation formed an improved anaerobic environment for the methanogenic consortia and improved the dissolution of organic compounds in the liquid. As observed by microscopy, the consortium bacteria showed increased growth in nutrition solution #2, implying that the DOL emulsion helped maintain a balance in the growth of the methanogenic consortia.

### pH changes with methane yield

**Fig 8:**
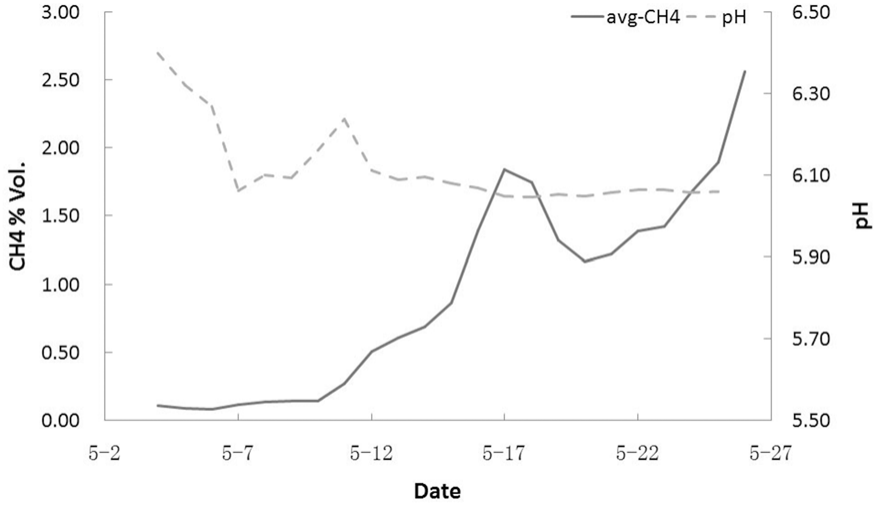
pH changes during the methane yield process in nutrition solution #1. The pH decreased with increasing methane yield rate.

**Fig 9:**
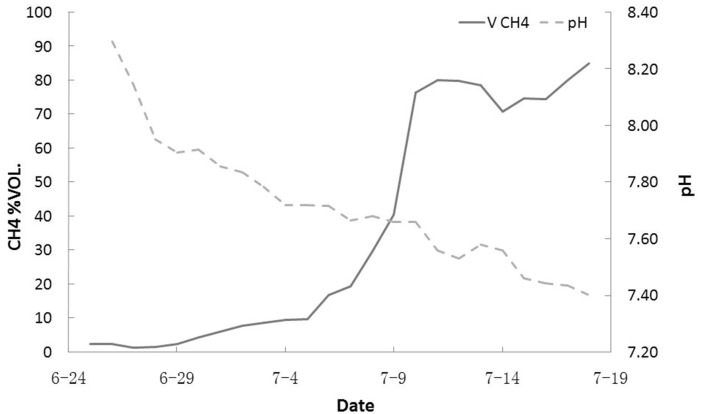
pH changes during the methane yield process in nutrition solution #2. The pH decreased with increasing methane yield rate.

pH is an important factor influencing the environmental conditions and metabolism of the methanogenic consortium. A lower pH can help achieve a higher methane yield rate more rapidly. Hydrogen ions are generated during the bacterial metabolic processes of hydrolytic and fermentative bacteria. A higher concentration of hydrogen ions may provide a more suitable and favorable culture environment. The pH experiment showed that the community had the ability and tendency to alter the culture environment (Figs 8 and 9). In both the #1 and #2 culture experiments, the pH decreased when the methane yield rate increased. In the #2 experiment, this phenomenon was more pronounced. Over 23 days, the pH dropped from 8.21 to 7.40.

In these experiments, all solutions and equipment were autoclaved except for the formation water and coal samples. Microscopy tests for six formation water samples showed that no living microbial organisms were present. The only sources for the microbial community were the coal samples.

## Discussion and Conclusions

This research examined the efficacy of DOL emulsion nutrition for improving methanogenic consortia biomethane production. The yields of methane and carbon dioxide were monitored in real time. The effects of pH on methanogenic consortia were also examined. Studies on the influence of DOL emulsions showed that the methane yield is variable and dependent on the stability of the methanogenic consortia. The results of this study revealed the following: (1) The DOL emulsion improved the biomethane yield compared with the control experiment. The highest methane production rate achieved was 40.32 mL/g•day^-1^. (2) Real-time monitoring of the methane yield indicated that the coalbed methanogenic microbial fermentation process was not stable but could be continuously maintained when the microbial cultures were kept in balance. (3) Carbon dioxide was an important intermediate in the coal fermentation process and the main carbon source of methanogens. It is important to measure the concentration of carbon dioxide in mixed gas as it affects the stability of methanogenic consortia. The carbon dioxide concentration could be kept low when the methanogenic and hydrolytic bacteria consortia were in a balanced condition. (4) Methanogenic consortia have the ability and tendency to alter the culture environment. pH 6.10 was the optimum pH in this experiment. These experiments were simple but very important for the feature research of MECoM. In some cases, methanogenic bacteria are inhibited because of nutritional deficiency. The modification of the coalbed microbial environment is an important method for maintaining the microbial consortia in a good condition. Controlling the available nutrients is necessary for improving the effective carbon source availability and decreasing the carbon dioxide concentration in mixed gas. DOL emulsion nutrition is an effective method for improving the nutritional capacity of coal. To fully understand the effect of DOL emulsions in enhancing the methane yield of methanogenic consortia, further study is required. There are three mechanisms that could be responsible for the effects of DOL. (1) DOL could adsorb at the coal surface (Fig 7). This would assist anaerobic fermentation in the cleat system in coal. (2) DOL could improve the dissolution of the organic compounds of coal, and it could improve the nutrition availability of coal for microbials. (3) Some DOL compounds might provide nutrients for certain bacteria and improve the microbial balance between methanogenic and hydrolytic bacterial consortia. Determining which of these mechanisms is ultimately responsible requires more research.

Real-time monitoring was used in this study. The experimental data revealed that the methane biosynthesis rate and carbon dioxide yield rate need further study. Methane biosynthesis was not a stable process. The data demonstrated that coalbed methanogenic bacterial cooperation may have a periodic nature. Exploring the mechanism of this periodicity could improve research into coalbed methanogenic consortia ecology. Carbon dioxide is an important factor that governs microbial group balance. Further study on this correlation is needed.

## References

[1] ARCTECH, Inc. Biological gasification of coals. In: Final report prepared for the Department of Energy under contract no. DE-AC21_87MC23285: DOE/MC/23285-2878 (DE90015337). 1990; pp. 130–135.

[2] ARCTECH, Inc. Biogasification of low-rank coals. In: Final report prepared for the Electric Power Research Institute. EPRI TR-101572. 1993; pp. 36–43.

[3] Barik S, Isbister J, Hawley B, Forgacs T, Reed L, Anspach G, et al Microbial conversion of coals to clean fuel forms. Prepr Pap Am Chem Soc Div Fuel Chem. 1998; 33: 548–553.

[4] Barik S, Isbister J, Harding R, Berrill P. Proceedings of the Second International Symposium on the Biological Processing of Coal. Palo Alto, CA: Electric Power Research Institute 1998; pp 23–38.

[5] Barik S, Tiemens K, Isbister J. Investigation of metabolites and optimization of bacterial culture for coal biogasification Phase 1 In: Topical report, February 1989-December 1990. Alexandria, VA (United States): ARCTECH, Inc. 1991; pp. 193–221.

[6] Scott AR. Improving coal gas recovery with microbially enhanced coalbed methane In: Coalbed Methane: Scientific, Environmental and Economic Evaluation. 1990; pp. 89–110.

[7] McIntosh J, Martini A, Petsch S, Huang R, Nüsslein K. Biogeochemistry of the Forest City Basin coalbed methane play. Int J Coal Geol. 2008; 76: 111–118.

[8] Thiele JH, Zeikus JG. Control of interspecies electron flow during anaerobic digestion: significance of formate transfer versus hydrogen transfer during syntrophic methanogenesis in flocs. Appl Environ Microbiol. 1988; 54: 20–29. 16347526 10.1128/aem.54.1.20-29.1988 PMC202391

[9] Winfrey MR. Microbial production of methane In: Altas RM, editor. Petroleum microbiology. New York: Macmillan 1984; pp. 153–220.

[10] Jones EJP, Voytek MA, Warwick PD, Corum MD, Cohn A, Bunnell JE, et al Bioassay for estimating the biogenic methane-generating potential of coal samples. Int J Coal Geol. 2008; 76: 138–150.

[11] McInerney MJ, Beaty PS. Anaerobic community structure from a nonequilibrium thermodynamic perspective. Can J Microbiol. 1988; 34: 487–493.

[12] Gateway Mine. Peabody Energy, 2014; Available: http://www.peabodyenergy.com /content/267/Publications/Fact-Sheets/Gateway-Mine.

[13] Tanner RS. Cultivation of bacteria and fungi In: Hurst CJ, Crawford RL, Knudsen GR, McInerney MJ, Stetzenbach LD, editors Manual of environmental microbiology, 2nd ed. Washington, D.C.: Australian Society for Microbiology (American Society for Microbiology) Press 2002; pp. 62–70.

[14] Faison B, Crawford D. The chemistry of low rank coal and its relationship to the biochemical mechanisms of coal biotransformation In: Crawford DL, editor. Microbial transformations of low rank coals. Boca Raton, FL: CRC Press pp. 1–26.

[15] Patrick CG, Feorge WS. Making microbial methane work: the potential for new biogenic gas. World Oil. 2007; 228: 37–48.

[16] Martini AM, Walter LM, Budai JM, Ku TCW, Kaiser CJ, Schoell M. Genetic and temporal relations between formation waters and biogenic methane: upper Devonian Antrim Shale, Michigan basin, USA. Geochim Cosmochim Acta 1998; 62: 1699–1720.

[17] Green MS, Flanegan KC, Gilcrease PC. Characterization of a methanogenic consortium enriched from a coalbed methane well in the Powder River Basin, U.S.A. Int J Coal Geol. 2008; 76: 34–45.

[18] Olson GJ, Brinckman F, Iverson W. Processing of coal with microorganisms Final report. Washington, DC (USA): National Bureau of Standards 1986.

